# Fructooligosaccharides (FOS) differentially modifies the *in vitro* gut microbiota in an age-dependent manner

**DOI:** 10.3389/fnut.2022.1058910

**Published:** 2023-01-12

**Authors:** Karley K. Mahalak, Jenni Firrman, Adrienne B. Narrowe, Weiming Hu, Steven M. Jones, Kyle Bittinger, Ahmed M. Moustafa, LinShu Liu

**Affiliations:** ^1^Dairy and Functional Foods Research Unit, Eastern Regional Research Center, Agricultural Research Service, United States Department of Agriculture, Wyndmoor, PA, United States; ^2^Division of Gastroenterology, Hepatology, and Nutrition, The Children’s Hospital of Philadelphia, Philadelphia, PA, United States; ^3^Department of Pediatrics, Perelman School of Medicine, University of Pennsylvania, Philadelphia, PA, United States

**Keywords:** Fructooligosaccharides, gut microbiome, *in vitro*, *Bifidobacterium*, prebiotics, *Lachnospiraceae*

## Abstract

**Introduction:**

Fructooligosaccharides (FOS) are well-known carbohydrates that promote healthy gut microbiota and have been previously demonstrated to enhance levels of *Bifidobacterium* and *Lactobacillus*. Its bifidogenic properties are associated with positive health outcomes such as reduced obesity and anti-inflammatory properties, and, therefore, is in use as a prebiotic supplement to support healthy gut microbiota. However, the gut microbiota changes with age, which may lead to differential responses to treatments with prebiotics and other dietary supplements.

**Methods:**

To address this concern, we implemented a 24-h *in vitro* culturing method to determine whether FOS treatment in three different adult age groups would have a differential effect. The age groups of interest ranged from 25 to 70 years and were split into young adults, adults, and older adults for the purposes of this analysis. Metagenomics and short-chain fatty acid analysis were performed to determine changes in the structure and function of the microbial communities.

**Results:**

These analyses found that FOS created a bifidogenic response in all age groups, increased overall SCFA levels, decreased alpha diversity, and shifted the communities to be more similar in beta diversity metrics. However, the age groups differed in which taxa were most prevalent or most affected by FOS treatment.

**Discussion:**

Overall, the results of this study demonstrate the positive effects of FOS on the gut microbiome, and importantly, how age may play a role in the effectiveness of this prebiotic.

## Introduction

The gut microbiota is an important factor to consider in general wellness and disease. It is well known that a healthy gut microbial community functions to aid against inflammation, gastrointestinal disorders such as irritable bowel disease and ulcerative colitis, diabetes, and obesity to name a few (1–4). Diet plays an important role in shaping the gut microbial community in terms of structure and function, and subsequently overall health of the host (2, 5, 6). The gut microbiota also changes with age (7–10). As humans age, certain members of the gut microbiome have been found to become more dominant, including *Bacteroides*, *Alistipes*, and *Parabacteroides* (11, 12). It has also been demonstrated that, in healthy individuals, the overall uniqueness of the gut microbial community increases with age and this pattern continues in the elderly population as long as those individuals remain healthy (11). However, the aging gut microbiome has also been shown to become less diverse and is also less stable, likely due to other age-related health factors (13). Aging is also associated with a decrease in the abundance of beneficial *Bifidobacterium sp.* (8, 10). A possible method to increase *Bifidobacterium sp*. and the abundances of other beneficial bacterial species is to include prebiotics, such as Fructooligosaccharides (FOS), as a regular dietary intervention (14, 15).

Fructooligosaccharides are common carbohydrates that are found in many vegetables, such as onions, chicory root, and garlic (16). They are also used to add non-digestible carbohydrates (NDC) to processed foods and as an alternative sweetener (17). The FOS are marketed as prebiotics that promote gut health because they are undigested until they reach the colon, where they are then metabolized by the gut microbiota (14). The fermentation of FOS and other prebiotics by the gut microbiota encourages the growth and health of the gut microbial community (14, 18, 19). The use of FOS as a dietary supplement has been associated with an increase in the abundance of the *Bifidobacterium* genus, which is an important member of the gut microbial community starting from birth and continuing onward throughout the aging process (8, 20). This bifidogenic property of FOS when used as a prebiotic has been demonstrated by several recent studies involving human participants (21–23). The changes to the gut microbial community caused by ingestion of FOS also cause changes in the metabolic products of that community, especially the production of short-chain fatty acids (SCFAs). SCFAs are the driver of many of the beneficial health effects associated with the gut microbial community including glucose homeostasis, the integrity of the gastrointestinal tract, and host immunity (24, 25).

Many studies related to FOS focus on its health benefits overall as a general prebiotic. However, as the gut microbiome field evolves, it has become increasingly clear that gut microbiomes are unique to each individual, which poses a problem as to which supplements or prebiotics may be most useful for any one individual. It is also well known that gut microbiomes change with age, which further complicates the issue of which prebiotics may be best for a person to use. To address the issue of age-related effects, we cultured gut microbial communities from six individuals of three adult age groups [young adult (25–35 year), adult (36–50 year), and older adult (51–70 year)] for 24 h with 5 g/L of FOS to understand first, how FOS may change the microbial communities overall, and second, how it may change the communities based on age. The dosage of FOS was decided based on previous work performed both *in vivo* and *in vitro*, similar to a reasonable amount of fiber supplementation in a day (26–29). To do so, the cultures were subjected to 16S rRNA sequencing analysis, qPCR analysis, and gas chromatography-flame ionizing detection to determine changes in their structure and function. Taken together, we found that FOS does cause changes in the structure and function of the gut microbial community in all age groups with notable age-dependent differences.

## Materials and methods

### *In vitro* culturing experiments

Fecal samples were obtained from 18 adults in 3 age groups with 6 individuals for each group, young adult (25–35 years of age), adult (36–50 years of age), and older adult (51–70 years of age). All donors were screened for adverse health conditions before use in this experiment. Exclusion criteria included any GI disorders, current medication, pregnancy, or lactation. Donors that were included had a BMI <30, were non-smokers, and had not taken any probiotics, prebiotics, or antibiotics for 3 months at a minimum. Once collected, the fresh feces collections were homogenized in a phosphate buffer containing 8.8 g/L K_2_HPO_4_; 6.8 g/L KH_2_PO_4_; 0.1 g/L sodium thioglycolate; and 0.015 g/L sodium dithionite to create a fecal slurry under anaerobic conditions as described previously (27, 28). This fecal slurry was used to inoculate 2 small cultures under anaerobic conditions, one control, and one containing 5 g/L Fructooligosaccharides (FOS) from chicory root (Sigma Aldrich, Saint Louis, MO, USA). The basal nutritional media used for both conditions contained the following commercially available ingredients: 16.3 g/L KH_2_PO_4_, 5.2 g/L K_2_HPO_4_, 2.0 g/L Yeast Extract, 2.0 g/L peptone, 2.0 g/L NaHCO_3_, 2.0 mL/L Tween80, 1.0 g/L mucin, 0.5 g/L L-cysteine and was made to a pH of 6.5. The inoculated cultures were grown for a total of 24 h and kept at 37°C as described previously (27). Samples were harvested from each culture at hours 0 (pre-treatment), 6, and 24 h. pH was monitored through the experiment.

### Gas Chromatography-Flame Ionizing Detection analysis of short-chain fatty acids

Samples of the communities were harvested at 0-, 6-, and 24-h post-inoculation and subject to analysis for the abundance of short-chain fatty acids (SCFA) and gas levels in the reactors. SCFA analysis was performed as described previously using the GC-2014 gas chromatography (Shimadzu) instrument (30). Detected SCFAs included: propionate, butyrate, acetate, valerate, isobutyrate, isovalerate, and isocaproate. Total SCFAs were calculated through summation of all SCFA levels, and total branch-chained SCFAs (BCFAs) were calculated through summation of isobutyrate, isovalerate, and isocaproate.

### qPCR analysis of *Bifidobacterium*

Deoxyribonucleic acid was extracted from a 1 mL volume of the microbial community that had been pelleted down using a fast DNA spin kit for soil (MP Biomedical). The abundance of the *Bifidobacterium* genus was determined using qPCR as described previously (31, 32). Integrated DNA Technologies (IDT) was our source of Primers and G-blocks that were used for standards. These standards were run using 10× serial dilutions from 1 × 10^7^–1 × 10^2^ copies/μL. The extracted DNA was diluted 100× in qPCR grade water (Roche). Samples were run in triplicate. Primers for this analysis were as follows: forward Bif243F 5′-TCGCGTCYGGTGTGAAAG-3′ and reverse Bif243R 5′-CCACATCCAGCRTCCAC-3′ (31). The conditions for qPCR analysis were as follows: 95°C for 5 m, followed by 40 cycles of 95°C for 15 s, 64°C for 15 s, and 72°C for 30 s, and ended with 83°C for 15 s accompanied by a melting curve analysis. Results were analyzed using the Roche Lightcycler software following the manufacturers guidelines to obtain absolute quantification of *Bifidobacterium*.

### Amplicon sequencing

16s rRNA sequencing was performed on the V1-V2 variable region of the 16S rRNA gene for microbial analysis. PCR reactions containing 0.5 μM of the primers, 0.34 U Q5 High-Fidelity DNA Polymerase (New England Biolabs), 0.2 mM dNTPs, and 2.5 μL of extracted DNA were carried out in duplicate. Cycling conditions included: 1 cycle of 98°C for 1 m; 25 cycles of 98°C for 10 s, 56°C for 20 s, and 72°C for 20 s; 1 cycle of 72°C for 8 m. Following the amplification step, PCR products were pooled and purified using SPRI beads. DNA was quantified using PicoGreen and pooled together in equal amounts. The Illumina MiSeq was used to sequence the final library using 2 × 250 bp chemistry.

### Bioinformatics and statistical analysis

Sequencing read QC and initial data processing was performed using QIIME2 (33). DADA2 was used to process read pairs to identify amplicon sequence variants (ASV) (34) and taxonomy was assigned using the naïve Bayes classifier implemented in scikit-bio (35) in comparison to the Greengenes references database (36). MAFFT was used to create a phylogenetic tree from the sequence data (37). Alpha diversity metrics were calculated using the phyloseq, ape, and picante packages in R (v. 4.1.3) (38) with helper functions from github/twbattaglia/btools (39–41). Beta diversity was calculated using the weighted UniFrac method (42, 43). Principle components analysis of SCFA data was performed in R using the “stats: prcomp” function with parameter scale = T and plotted using the “factoextra: fviz_pca_biplot” function (44). Visualizations were generated using the factoextra, vegan, tidyverse, ggplot2, pheatmap, and RColorBrewer R packages (44–48). Statistical analysis of differences by treatment or age group was performed using ANOVA with Tukey’s HSD *post-hoc* testing or multiple testing correction using the Benjamini–Hochberg method. Metagenomic compositions of samples were estimated using PICRUSt2 (49).

## Results

### FOS treatment increases short-chain fatty acid accumulation

**FIGURE 1:**
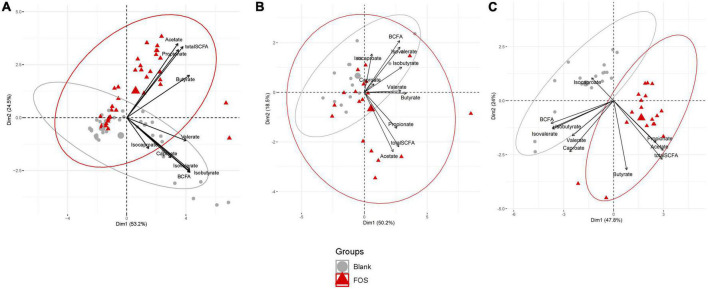
Fructooligosaccharides (FOS) supplementation induces a clear shift in short-chain fatty acid (SCFA) accumulation. Principal component analysis (PCA) analysis of metabolite data. **(A)** PCA plot includes all timespoints **(B)** 6 h timepoint **(C)** 24 h timepoint. For all PCA plots: Data are scaled (z-scored for each metabolite/measure) and ellipses represent a 95% interval for a normal distribution. The larger points represent the mean value for each group.

Gas Chromatography-Flame Ionizing Detection (GC-FID) was applied to determine the abundance of short-chain fatty acids (SCFAs), which is well-regarded as a healthy measure of gut microbiota functionality. This data was analyzed using principal component analysis (PCA) to determine the effect of FOS on SCFAs that are commonly produced by the gut microbiota (Figure 1). In Figure 1A, we illustrated this point using all time points and visualized a clear divergence of FOS-treated samples (red) from the untreated control samples (gray). When these results were separated by timepoint (Figures 1B, C), it became clear that SCFA accumulation was not immediate, meaning we observed little difference yet at 6 h post-treatment, but we did observe this significant and large change 24 h post-treatment.

**FIGURE 2:**
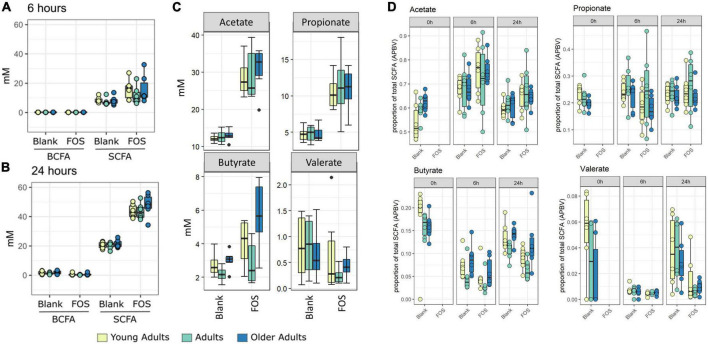
Fructooligosaccharides (FOS) addition stimulates short-chain fatty acid (SCFA) rather than branch-chained SCFA (BCFA) accumulation in fecal incubations. Concentration of SCFA measured by GC-FID. Branched chain fatty acid concentration (BCFA) compared with short-chain fatty acid (SCFA) concentration at 6 h **(A)** and 24 h **(B)**. For all age groups, FOS-treated samples have significantly higher levels of SCFA than control (*p* < 0.001, ANOVA, Tukey *post-hoc* test) **(A,B)**. **(C)** Shows individual SCFA concentrations of acetate, propionate, butyrate, and valerate. FOS-treated samples have significantly higher levels of acetate and propionate for all age groups, and butyrate for older adults only. (*p* < 0.008, ANOVA, Tukey *post-hoc* test). **(D)** Shows proportion of those individual SCFAs compared with total SCFA.

In Figure 1C, the control communities cluster to the left of the PCA plot, driven by levels of branched-chain fatty acids (BCFAs) and specifically of isobutyrate, isocaproate, caproate, valerate, and isovalerate. With the addition of FOS, we observe a shift to the right of this plot, driven by total SCFA accumulation, specifically of butyrate, acetate, and propionate. This difference in BCFA and SCFA accumulation, meaning the increase in overall SCFA accumulation and no present increase in BCFA accumulation, is apparent in bar plots in Figure 2. In fact, BCFA levels are greater in control samples when compared with FOS-treated samples after 24 h of incubation, though this difference is not significant. These changes in SCFA accumulation do not change with age group. When broken down by age group, we found that butyrate was the only SCFA that was different between age groups (Figure 2C). Butyrate increased in all age groups, but it increased the least in the adult age group, and it had the greatest concentration in the older adult age group. We also compared proportions of those four key SCFA to total SCFA concentrations in Figure 2D. In this part of the analysis, we found that increases in acetate, propionate occur with FOS treatment, even proportionally. However, butyrate and valerate did not increase with FOS treatment.

### FOS treatment had a distinct effect on gut microbial community diversity

**FIGURE 3:**
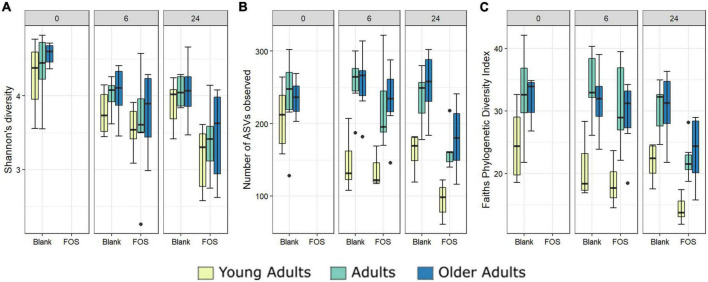
Alpha diversity differs significantly with Fructooligosaccharides (FOS) treatment and with donor age. Three alpha diversity measures shown; **(A)** Shannon’s Diversity **(B)** Number of ASVs observed, **(C)** Faith’s phylogenetic diversity index. Significance was determined using ANOVA with Tukey *post-hoc* test. Significance in all 3 at 24-h post inoculation with *p* < 0.001 in all cases. For **(B,C)**, alpha diversity also differs significantly by age group (*p* < 0.001).

In addition to the functional analysis performed by way of SCFA accumulation, we analyzed changes to the microbial communities in response to FOS treatment in all three age groups using 16S rRNA marker gene sequencing. Alpha diversity was measured using three different methods, Shannon’s Diversity, species richness, and Faith’s phylogenetic diversity index (Figure 3). In all measurements of alpha diversity, changes in the community that occur between hour 0 and hour 6 are negligible. However, once the 24-h timepoint is reached, we observed a significant decrease across all measures of alpha diversity with the addition of FOS. This decrease in diversity shown by Shannon’s diversity metric does not differ by age group (Figure 3A). However, for both species richness (shown by the number of ASVs observed) and Faith’s phylogenetic diversity index measurements we found a significant difference in age groups driven by a much lower level of alpha diversity in the young adult group compared with both older adult groups (Figures 3B, C, yellow).

**FIGURE 4:**
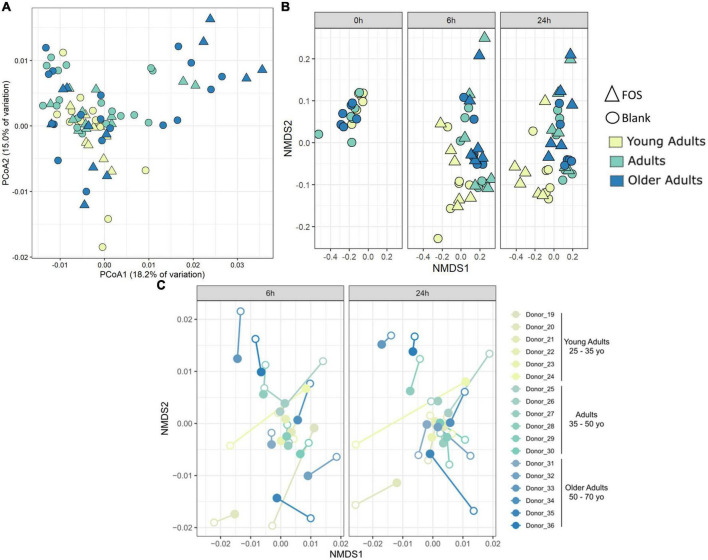
Community-level shifts in diversity are donor-specific and are minimal in a 24-h incubation. **(A)** Principal coordinates analysis of weighted UniFrac distance shows some divergence by age group but no clustering by treatment. **(B)** PICRUSt analysis using Bray-Curtis NMDS analysis. **(C)** Procrustes analysis on Bray-Curtis dissimilarity shows that most samples group by donor despite treatment. Procrustes analysis was significant *p* < 0.001 with matrix (treated/control) correlation of 0.6359. Filled circles are FOS-treated, open circles are controls. Same-donor pairs are connected by lines.

We also performed principal coordinate analysis (PCoA) of weighted UniFrac distances using 16S rRNA gene sequencing data (Figure 4). Figure 4A shows all time points and age groups together: there was slight divergence by age group, but no divergence by treatment. In Figure 4C, however, in the Procrustes analysis of Bray-Curtis dissimilarity, the chart is separated by communities at either 6 h of incubation or 24 h of incubation. In this figure, we show the control (blank) samples connected to the FOS-treated communities from the same donor and observed that most communities are grouped by the donor and not by treatment. What is interesting about this figure is that the FOS-treated samples (closed circles) appeared to be converging together in this measure of beta diversity compared with the untreated control, suggesting that the FOS-treated samples were becoming more similar over time.

To determine whether FOS affected the functional capacity of the community and not simply the phylogenetic composition, we used PICRUSt to infer both the functions and the abundance of those functions within the gut microbial communities. Repeated beta diversity analysis using the abundances of estimated KEGG functions in place of ASV abundances demonstrated that the young adult group clusters apart from the two other age groups in terms of function (Figure 4B), which is different from what is seen in Figure 4A with phylogenetic composition alone. There is no significant difference here in terms of FOS treatment compared with control. This indicates that while FOS is changing the microbial community phylogenetically, it is not changing the functional capacity of this community.

### FOS significantly decreased the abundance of specific taxa

**FIGURE 5:**
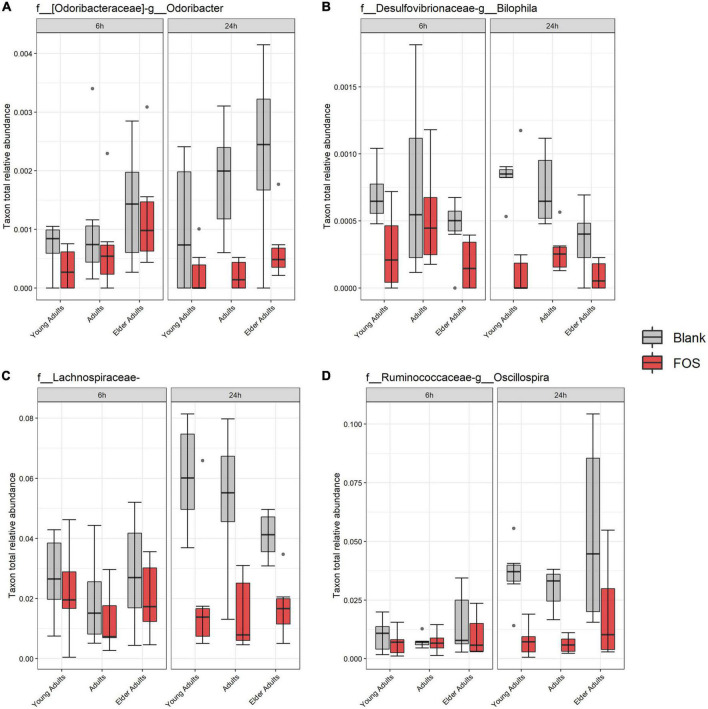
Significant taxa by treatment. Relative abundance of these taxa were determined by 16S rRNA sequencing. Significance was determined using ANOVA with Tukey’s HSD *post-hoc* testing. **(A)** *Odoribacter*; **(B)** *Bilophila*; **(C)** *Lachnospiraceae* of unclassified genus; **(D)** *Oscillospira*.

Using the 16S rRNA sequencing data, we also searched for specific bacterial taxa that changed in relative abundance due to treatment with FOS. We identified only four members of the microbial communities whose abundance significantly decreased in response to FOS treatment, despite their low abundance in the communities (Figure 5). The first was genus *Odoribacter*, which decreased in relative abundance when treated with FOS in the adult and older adult age groups, but it did not significantly decrease in abundance in the young adult age group. The second taxa member was genus *Bilophila*, which decreased significantly in all age groups after 24 h of incubation with FOS. Unclassified members within the family *Lachnospiraceae* were the third member of the taxa that significantly decreased in response to FOS. Genus *Oscillospira* was the final member of the taxa that significantly decrease in abundance in the communities treated with FOS. In this case, it was most prevalent in young adults and adults. *Oscillospira* did decrease in abundance in the older adult age group, but there was some overlap between the control and treatment groups.

**FIGURE 6:**
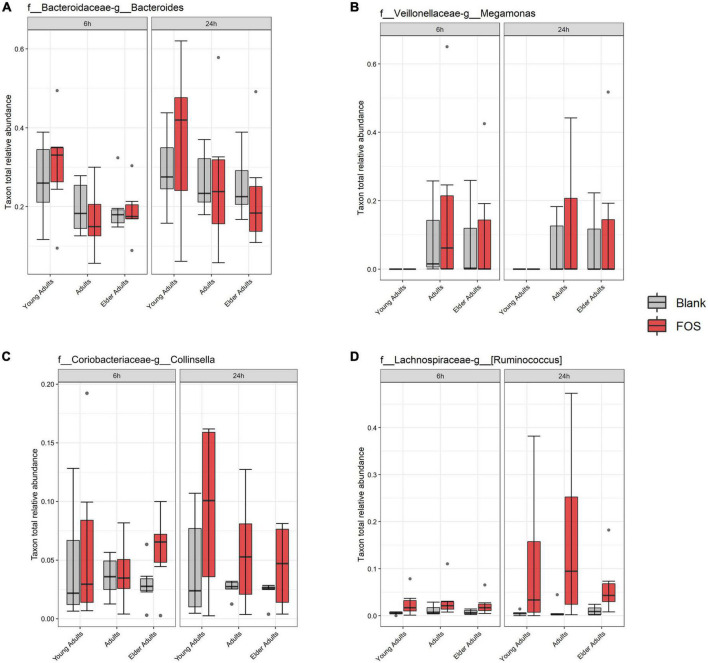
Most abundant taxa in the Fructooligosaccharides (FOS)-treated group. Relative abundance of these taxa were determined by 16S rRNA sequencing. Significance was determined using ANOVA with Tukey’s HSD *post-hoc* testing. **(A)** *Bacteroides*; **(B)** *Megamonas*; **(C)** *Collinsella*; **(D)** *Ruminococcus*.

While the overall relative abundance of the above taxa that significantly decreased in the community with FOS treatment was small, there was the possibility that gaps are left in community function by their absence. To explore this idea, we found taxa that were in the highest abundance in the FOS-treated groups, particularly those that were increased at 24 h of incubation. This group, shown in Figure 6, included members that were expected to be highly abundant, such as *Bacteroides*, *Megamonas, Collinsella*, and *Ruminococcus*. Each of these members increased in abundance with FOS treatment, though that increase was not statistically significant. Two of these community members increased in an age-group-dependent manner. *Bacteroides* increased in abundance in the young adult group only. *Collinsella* and *Ruminococcus* increased in abundance in all age groups. However, *Megamonas* did not increase in abundance in young adults (although it appears to be nearly non-existent in the young adult microbial communities) but did increase in both the adult and elder-adult age groups.

### *Bifidobacterium* abundance increased significantly with FOS treatment

**FIGURE 7:**
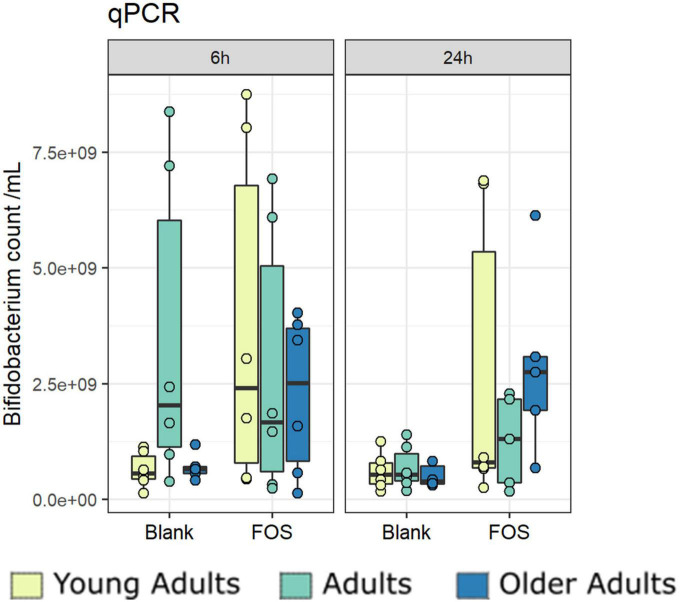
*Bifidobacterium* increase significantly with Fructooligosaccharides (FOS) treatment. Absolute abundances of *Bifidobacterium* increase with FOS treatment using qPCR.

Due to primer mismatches, the 16S rRNA target gene sequence analysis as performed using V1/V2 regions for the overall community analysis above does not accurately detect taxa within *Bifidobacteriales.* However, *Bifidobacterium* abundance is of particular interest when addressing the effect of FOS on the gut microbiota (50). To address this issue, we performed a qPCR analysis targeting *Bifidobacterium* to find whether the addition of FOS impacted abundance. After 24 h of incubation, there was an increase in *Bifidobacterium* across all age groups (Figure 7). The increase found in young adults was the least consistent across donors. The adult age group also showed a real, but not statistically significant increase in *Bifidobacterium* in some of the donors. The older adult group, however, has the largest increase in *Bifidobacterium* which was also statistically significant. This is an important finding, because *Bifidobacterium* is known to decrease with age, is associated with good health of the gut microbiota, and has been demonstrated to support a proper immune system (8, 15, 51).

### SCFA abundance correlated with taxa abundance gives insight into FOS metabolism

**FIGURE 8:**
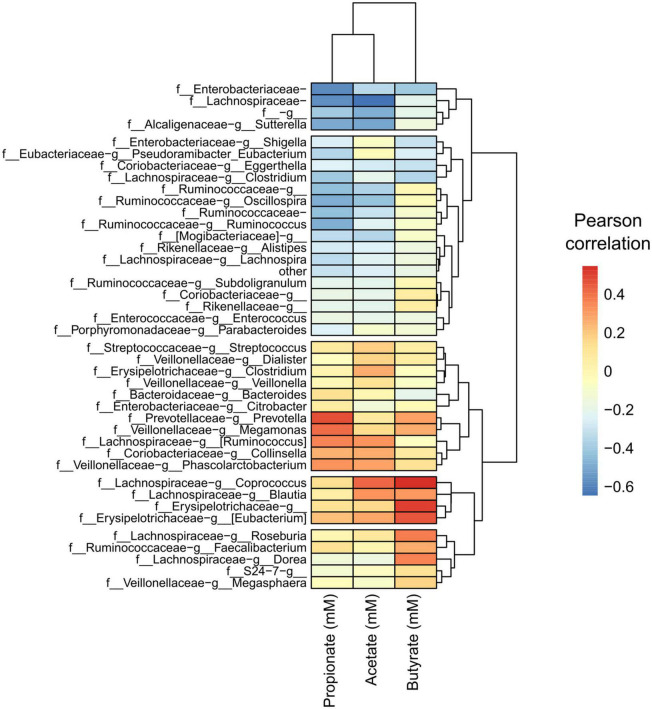
A conserved group of taxa correlated with short-chain fatty acid (SCFA) concentrations, with apparent specialization by taxon. Heatmap of Pearson correlations of taxa (family-genus) with SCFA concentrations and gas production. Only taxa having an average relative abundance of at least 0.1% across all samples are included. Samples and metabolites are clustered hierarchically.

Next, we performed a Pearson correlation of identified taxa with SCFA concentrations to find any specialization of SCFA correlation within taxa. We detected a pattern with specific taxa and the most abundant SCFAs found in the cultured gut microbiome communities, acetate, propionate, and butyrate (Figure 8). We found a clustered group of taxa that were negatively correlated with these SCFAs as well as in gas concentration. The taxon with the largest negative correlation was *Lachnospiraceae* (of unclassified genus), which was also a taxon that significantly decreased in response to FOS. Other community members that were negatively correlated with SCFA accumulation included: *Enterobacteriaceae* (of unclassified genus), *Ruminococcaceae* (of unclassified genus), and *Lachnospiraceae Clostridium*. Many taxa had a positive correlation with SCFA concentration, including some specific *Lachnospiraceae* members, such as *Blautia*, *Coprococcus*, and *Ruminococcus*. *Collinsella* also had a positive correlation with all SCFAs shown in Figure 8, which was identified in Figure 6 as a member that increased with the addition of FOS.

**FIGURE 9:**
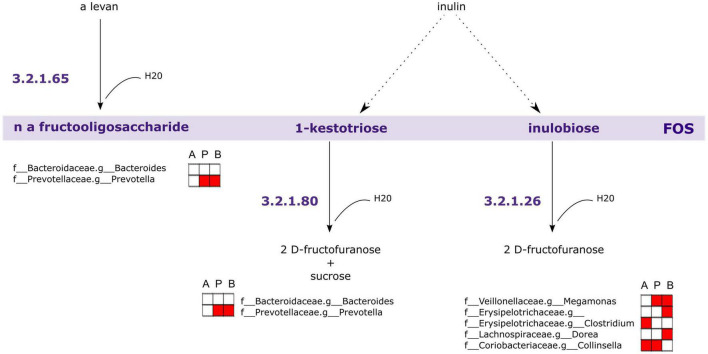
Genes associated with Fructooligosaccharides (FOS) metabolism are encoded within taxa correlated with short-chain fatty acid (SCFA) concentrations. PICRUSt2 analysis of 16S rRNA indicates taxa likely to possess genes involved in FOS and inulin metabolism. The largest contributors of these genes in the samples are those taxa associated with SCFA concentrations. A, acetate, P, propionate, B, butyrate. Red fill indicates Pearson correlation ≥ 0.2.

Following this analysis, we performed a PICRUSt2 analysis of the 16S rRNA sequencing data to discover genes that are associated with FOS metabolism. In Figure 9, we illustrated a suggested pathway involved in FOS and inulin metabolism, including which members of the gut microbial community found in this study may be involved in each step. In the figure, red indicates the bacteria that are positively correlated with SCFA concentrations in Figure 8. The results of our analysis indicated that genus *Bacteroides* and genus *Prevotella* may be involved in the metabolism of FOS and inulin to 1-kestotriose. There are more taxa with genes associated with the metabolism of inulin to inulobiose present in these communities, however, including genus *Megamonas, Erysipelotrichaceae* of unclassified genera, genus *Clostridium*, genus *Dorea*, and genus *Collinsella.* Of these, *Megamonas*, *Erysipelotrichaceae*, and *Dorea* are positively correlated with butyrate, *Clostridium* and *Collinsella* are positively correlated with Acetate, and *Megamonas* and *Collinsella* are positively correlated with propionate.

## Discussion

Diet and nutrition are important factors that impact the gut microbiota, which in turn impacts human health. Over the past several decades, the diets available around the world have changed, some of which are positive, for example, greater nutritional availability in terms of energy available from foods and more diverse options due to global commerce (52). Other aspects of these changes can adversely affect health, such as an increase in calorie-dense food with low nutritional value and an increase in highly processed foods like sweetened beverages (21). Those changes, combined with an increasingly sedentary lifestyle are just a few of the many factors that are associated with poor overall health (21, 53). These dietary shifts have corresponded to some general trends in the gut microbial population, including decreases in bacterial diversity that are often associated with poor health outcomes. Recent studies in humans have found that the western diet decreases gut microbial diversity with a subsequent decrease in the range of mono- and polysaccharides that can be digested by the gut microbiota (54, 55). A recent study in rats found that the addition of a western diet rapidly changed the gut microbiome to a state of dysbiosis and increased markers of inflammation (56). Taken together, this phenomenon helps to explain the current interest in improving gut microbiota health and the interest in prebiotics. FOS in particular is well-known to have bifidogenic effects and to increase SCFA abundance in the gut microbiota, which has made it a popular choice as a prebiotic.

In this study, we found that treatment of the gut microbial communities with FOS created a clear shift in SCFA accumulation compared with control after 24 h of incubation. We also found that BCFA accumulation was not significantly affected by this treatment, though there was a slight decrease with FOS treatment. This is similar to a previous finding that FOS significantly decreases BCFA abundance after at least 48 h of treatment (26). BCFAs have been found to increase with more protein-heavy diets and decrease with the addition of complex carbohydrates (57). It may be that given more time the accumulation of BCFA would have decreased significantly in the FOS treatment group compared with control. This study illustrated that the high abundance SCFAs, including acetate, propionate, and butyrate are highly influenced by the addition of this prebiotic. This is in keeping with other findings of *in vitro* batch culture experiments using FOS treatment (26–29). However, it is in contrast with a recent finding from a clinical trial where FOS decreased the amount of butyrate in fecal microbiome samples after 14 days of treatment (58). It is possible that *in vivo* the excess butyrate produced is used by other intestinal tissue or other members of the gut microbial community that are removed or unable to be cultured *in vitro*. The overall changes in SCFA abundance were not different between age groups. The exception to this was butyrate, which was higher in abundance in the older adults group compared with the young adult and adult groups and was increased the least in the adult age group.

The results of our genomic analyses showed that FOS decreased alpha diversity across all measures and all age groups, but this change was most pronounced in the young adult group. This finding is in contrast with a recent *in vivo* study that found FOS had no impact on the alpha diversity of young adults (58). In previous work with young adults and FOS, researchers found that FOS increased the *Bifidobacteria* present but decreased the butyrate production in young people (35, 58). Conversely, in this study, the abundance of *Bifidobacteria* did increase with FOS treatment, in conjunction with an increase in the production of beneficial butyrate. This study also found that the addition of FOS to the microbial communities caused a shift to make them more similar to each other after 24 h, regardless of age group. When analyzing beta diversity using functional analysis instead of phylogenetic analysis, however, it was discovered that FOS did not change the functionality of the communities, but that the young adult group did cluster away from the adult and older adult groups.

Next, four taxa were identified that significantly decreased in response to FOS treatment. The first was genus *Odoribacter*, which has also been found to decrease in response to daily orange juice consumption and increase in patients with cognitive impairment (59, 60). The second was *Bilophila*, which has been found to promote the production of lipopolysaccharides (LPS), the overproduction of which is associated with negative health outcomes, such as inflammation and obesity (4, 61). *Bilophila* has also been found to decrease in abundance in mice when their diets were supplemented with adzuki bean (62).

Members of the *Lachnospiraceae* family that were otherwise unclassified by our methods were also found to decrease in response to FOS, which is a well-studied, complex family of microorganisms whose many members are known to have a great impact on gut health, however, since this grouping is not identified we will not speculate further on their role (63).

Overall, we found that several abundant taxa were correlated with SCFA analysis and known to carry genes that are involved in FOS metabolism pathways. Some of the taxa that are associated with these pathways, especially the inulobiose pathway, are prevalent members of the community that increased in response to FOS (64). These members include *Collinsella*, *Megamonas*, and *Ruminococcus*, as well as *Bacteroides*. Of these, *Bacteroides* and *Collinsella* were greater in abundance in the young adult age group, whereas *Megamonas* was more prevalent in the adult and older adult age groups.

As a whole, this study found that FOS treatment modulated the gut microbiome community in a similar, but age-dependent manner. In all groups, we found an increase in SCFA production and abundance over 24 h with FOS treatment, a decrease in all measures of alpha diversity, and a converging of communities in beta diversity measurements. FOS treatment caused the expected bifidogenic effect in all age groups, with the largest increase in the older adult age group. After analysis of the microbial community compositions and their functions, we found that prevalent taxa in the communities are known participants in the metabolism of FOS, and their abundance is varied in the three age groups. Our findings indicate that FOS changes the gut microbial community, community changes are specific to age, and the function of the microbial community in terms of FOS metabolism is taken over by alternate, age-dependent taxa.

## Data availability statement

The raw sequencing data presented in this study are deposited in the NCBI Sequence Read Archive (SRA), accession number: PRJNA883714. The SCFA data can be found in the Supplementary material.

## Author contributions

KM contributed to the original draft writing, experimental design, and data analysis. JF contributed to the reviewing and editing of the manuscript, experimental design, and analysis. AN contributed to the reviewing and editing the manuscript, data, and statistical analysis. WH and KB contributed to the data analysis and reviewing and editing of the manuscript. SJ and AM contributed to the sequencing and data analysis as well as reviewing and editing the manuscript. LL contributed to the experimental design and reviewing and editing the manuscript. All authors contributed to the article and approved the submitted version.

## References

[1] Feng Q Chen W Wang Y. Gut microbiota: an integral moderator in health and disease. *Front Microbiol.* (2018) 9:151. 10.3389/fmicb.2018.00151 29515527 PMC5826318

[2] Baothman O Zamzami M Taher I Abubaker J Abu-Farha M. The role of gut microbiota in the development of obesity and diabetes. *Lipids Health Dis.* (2016) 15:108. 10.1186/s12944-016-0278-4 27317359 PMC4912704

[3] Al Bander Z Nitert M Mousa A Naderpoor N. The gut microbiota and inflammation: an overview. *Int J Environ Res Public Health.* (2020) 17:7618. 10.3390/ijerph17207618 33086688 PMC7589951

[4] Beaumont M Goodrich J Jackson M Yet I Davenport E Vieira-Silva S Heritable components of the human fecal microbiome are associated with visceral fat. *Genome Biol.* (2016) 17:189. 10.1186/s13059-016-1052-7 27666579 PMC5036307

[5] Wu G Chen J Hoffmann C Bittinger K Chen Y Keilbaugh S Linking long-term dietary patterns with gut microbial enterotypes. *Science.* (2011) 334:105–8. 10.1126/science.1208344 21885731 PMC3368382

[6] Power S O’Toole P Stanton C Ross R Fitzgerald G. Intestinal microbiota, diet and health. *Br J Nutr.* (2014) 111:387–402. 10.1017/S0007114513002560 23931069

[7] Induri S Kansara P Thomas S Xu F Saxena D Li X. The gut microbiome, metformin, and aging. *Annu Rev Pharmacol Toxicol.* (2022) 62:85–108. 10.1146/annurev-pharmtox-051920-093829 34449247

[8] Arboleya S Watkins C Stanton C Ross R. Gut *Bifidobacteria* populations in human health and aging. *Front Microbiol.* (2016) 7:1204. 10.3389/fmicb.2016.01204 27594848 PMC4990546

[9] Yatsunenko T Rey F Manary M Trehan I Dominguez-Bello M Contreras M Human gut microbiome viewed across age and geography. *Nature.* (2012) 486:222–7. 10.1038/nature11053 22699611 PMC3376388

[10] Rinninella E Raoul P Cintoni M Franceschi F Miggiano G Gasbarrini A What is the healthy gut microbiota composition? A changing ecosystem across age, environment, diet, and diseases. *Microorganisms.* (2019) 7:14. 10.3390/microorganisms7010014 30634578 PMC6351938

[11] Wilmanski T Diener C Rappaport N Patwardhan S Wiedrick J Lapidus J Gut microbiome pattern reflects healthy ageing and predicts survival in humans. *Nat Metab.* (2021) 3:274–86. 10.1038/s42255-021-00348-0 33619379 PMC8169080

[12] Claesson M Cusack S O’Sullivan O Greene-Diniz R de Weerd H Flannery E Composition, variability, and temporal stability of the intestinal microbiota of the elderly. *Proc Natl Acad Sci USA.* (2011) 108 (Suppl. 1):4586–91. 10.1073/pnas.1000097107 20571116 PMC3063589

[13] Jayanama K Theou O. Effects of probiotics and prebiotics on frailty and ageing: a narrative review. *Curr Clin Pharmacol.* (2020) 15:183–92. 10.2174/1574884714666191120124548 31750806

[14] Roberfroid M Gibson G Hoyles L McCartney A Rastall R Rowland I Prebiotic effects: metabolic and health benefits. *Br J Nutr.* (2010) 104:S1–63. 10.1017/S0007114510003363 20920376

[15] Toward R Montandon S Walton G Gibson G. Effect of prebiotics on the human gut microbiota of elderly persons. *Gut Microbes.* (2012) 3:57–60. 10.4161/gmic.19411 22555548

[16] Campbell J Bauer L Fahey G Hogarth A Wolf B Hunter D. Selected fructooligosaccharide (1-kestose, nystose, and 1F-β-fructofuranosylnystose) composition of foods and feeds. *J Agric Food Chem.* (1997) 45:3076–82. 10.1021/jf970087g

[17] Oku T Nakamura S. Fructooligosaccharide: metabolism through gut microbiota and prebiotic effect. *Food Nutr J.* (2017) 2:128. 10.29011/2575-7091.100028

[18] Gibson G. Dietary modulation of the human gut microflora using the prebiotics oligofructose and inulin. *J Nutr.* (1999) 129:1438S–41. 10.1093/jn/129.7.1438S 10395616

[19] Li H Zhou D Gan R Huang S Zhao C Shang A Effects and mechanisms of probiotics, prebiotics, synbiotics, and postbiotics on metabolic diseases targeting gut microbiota: a narrative review. *Nutrients.* (2021) 13:3211. 10.3390/nu13093211 34579087 PMC8470858

[20] Favier C Vaughan E De Vos W Akkermans A. Molecular monitoring of succession of bacterial communities in human neonates. *Appl Environ Microbiol.* (2002) 68:219–26. 10.1128/AEM.68.1.219-226.2002 11772630 PMC126580

[21] Tandon D Haque M Gote M Jain M Bhaduri A Dubey A A prospective randomized, double-blind, placebo-controlled, dose-response relationship study to investigate efficacy of fructo-oligosaccharides (FOS) on human gut microflora. *Sci Rep.* (2019) 9:5473. 10.1038/s41598-019-41837-3 30940833 PMC6445088

[22] Kato T Fukuda S Fujiwara A Suda W Hattori M Kikuchi J Multiple omics uncovers host–gut microbial mutualism during prebiotic fructooligosaccharide supplementation. *DNA Res.* (2014) 21:469–80. 10.1093/dnares/dsu013 24848698 PMC4195493

[23] Tuohy K Kolida S Lustenberger A Gibson G. The prebiotic effects of biscuits containing partially hydrolysed guar gum and fructo-oligosaccharides–a human volunteer study. *Br J Nutr.* (2001) 86:341–8. 10.1079/BJN2001394 11570986

[24] Bhattacharya T Ghosh T Mande S. Global profiling of carbohydrate active enzymes in human gut microbiome. *PLoS One.* (2015) 10:e0142038. 10.1371/journal.pone.0142038 26544883 PMC4636310

[25] Anand S Kaur H Mande S. Comparative in silico analysis of butyrate production pathways in gut commensals and pathogens. *Front Microbiol.* (2016) 7:1945. 10.3389/fmicb.2016.01945 27994578 PMC5133246

[26] Pham V Calatayud M Rotsaert C Seifert N Richard N Van den Abbeele P Antioxidant vitamins and prebiotic FOS and XOS differentially shift microbiota composition and function and improve intestinal epithelial barrier *in vitro*. *Nutrients.* (2021) 13:1125. 10.3390/nu13041125 33805552 PMC8066074

[27] Van den Abbeele P Taminiau B Pinheiro I Duysburgh C Jacobs H Pijls L Arabinoxylo-oligosaccharides and inulin impact inter-individual variation on microbial metabolism and composition, which immunomodulates human cells. *J Agric Food Chem.* (2018) 66:1121–30. 10.1021/acs.jafc.7b04611 29363966

[28] Van den Abbeele P Verstrepen L Ghyselinck J Albers R Marzorati M Mercenier A. A novel non-digestible, carrot-derived polysaccharide (cRG-I) selectively modulates the human gut microbiota while promoting gut barrier integrity: an integrated *in vitro* approach. *Nutrients.* (2020) 12:1917. 10.3390/nu12071917 32610452 PMC7400138

[29] Hajar-Azhari S Abd Rahim M Sarbini S Muhialdin B Olusegun L Saari N. Enzymatically synthesised fructooligosaccharides from sugarcane syrup modulate the composition and short-chain fatty acid production of the human intestinal microbiota. *Food Res Int.* (2021) 149:110677. 10.1016/j.foodres.2021.110677 34600679

[30] De Weirdt R Possemiers S Vermeulen G Moerdijk-Poortvliet T Boschker H Verstraete W Human faecal microbiota display variable patterns of glycerol metabolism. *FEMS Microbiol Ecol.* (2010) 74:601–11. 10.1111/j.1574-6941.2010.00974.x 20946352

[31] Rinttilä T Kassinen A Malinen E Krogius L Palva A. Development of an extensive set of 16S rDNA-targeted primers for quantification of pathogenic and indigenous bacteria in faecal samples by real-time PCR. *J Appl Microbiol.* (2004) 97:1166–77. 10.1111/j.1365-2672.2004.02409.x 15546407

[32] Orschler L Agrawal S Lackner S. On resolving ambiguities in microbial community analysis of partial nitritation anammox reactors. *Sci Rep.* (2019) 9:1–10. 10.1038/s41598-019-42882-8 31061389 PMC6502876

[33] Bolyen E Rideout J Dillon M Bokulich N Abnet C Al-Ghalith G Reproducible, interactive, scalable and extensible microbiome data science using QIIME 2. *Nat Biotechnol.* (2019) 37:852–7. 31341288 10.1038/s41587-019-0209-9 PMC7015180

[34] Callahan B McMurdie P Rosen M Han A Johnson A Holmes S. DADA2: high-resolution sample inference from Illumina amplicon data. *Nat Methods.* (2016) 13:581–3. 10.1038/nmeth.3869 27214047 PMC4927377

[35] Bokulich N Kaehler B Rideout J Dillon M Bolyen E Knight R Optimizing taxonomic classification of marker-gene amplicon sequences with QIIME 2’s q2-feature-classifier plugin. *Microbiome.* (2018) 6:90. 10.1186/s40168-018-0470-z 29773078 PMC5956843

[36] McDonald D Price M Goodrich J Nawrocki E DeSantis T Probst A An improved Greengenes taxonomy with explicit ranks for ecological and evolutionary analyses of bacteria and archaea. *ISME J.* (2012) 6:610–8. 10.1038/ismej.2011.139 22134646 PMC3280142

[37] Katoh K Standley D. MAFFT multiple sequence alignment software version 7: improvements in performance and usability. *Mol Biol Evol.* (2013) 30:772–80. 10.1093/molbev/mst010 23329690 PMC3603318

[38] R Core Team. *R: A language and environment for statistical computing.* Vienna: R Foundation for Statistical Computing (2022).

[39] Kembel S Cowan P Helmus M Cornwell W Morlon H Ackerly D Picante: R tools for integrating phylogenies and ecology. *Bioinformatics.* (2010) 26:1463–4. 10.1093/bioinformatics/btq166 20395285

[40] Paradis E Schliep K. ape 5.0: an environment for modern phylogenetics and evolutionary analyses in R. *Bioinformatics.* (2019) 35:526–8. 10.1093/bioinformatics/bty633 30016406

[41] McMurdie P Holmes S. phyloseq: an R package for reproducible interactive analysis and graphics of microbiome census data. *PLoS One.* (2013) 8:e61217. 10.1371/journal.pone.0061217 23630581 PMC3632530

[42] Lozupone C Knight R. UniFrac: a new phylogenetic method for comparing microbial communities. *Appl Environ Microbiol.* (2005) 71:8228–35. 10.1128/AEM.71.12.8228-8235.2005 16332807 PMC1317376

[43] Lozupone C Hamady M Kelley S Knight R. Quantitative and qualitative β diversity measures lead to different insights into factors that structure microbial communities. *Appl Environ Microbiol.* (2007) 73:1576–85. 10.1128/AEM.01996-06 17220268 PMC1828774

[44] Kassambara A Mundt F. *Factoextra: extract and visualize the results of multivariate data analyses. R package version 107.* (2020).

[45] Oksanen J Simpson G Blanchet F Kindt R Legendre P Minchin P *Vegan: community ecology package, R package version 2.6-2.* (2018).

[46] Wickham H Averick M Bryan J Chang W McGowan L François R Welcome to the Tidyverse. *J Open Source Softw.* (2019) 4:1686. 10.21105/joss.01686

[47] Wickham H. *Data analysis. ggplot2.* Berlin: Springer (2016). p. 189–201. 10.1007/978-3-319-24277-4_9

[48] Neuwirth E Neuwirth M. *Package ‘rcolorbrewer’ colorbrewer palettes R package version 1.1-2.* (2014).

[49] Douglas G Maffei V Zaneveld J Yurgel S Brown J Taylor C PICRUSt2 for prediction of metagenome functions. *Nat Biotechnol.* (2020) 38:685–8. 10.1038/s41587-020-0548-6 32483366 PMC7365738

[50] Chen Z Hui P Hui M Yeoh Y Wong P Chan M Impact of preservation method and 16S rRNA hypervariable region on gut microbiota profiling. *Msystems.* (2019) 4:e00271–18. 10.1128/mSystems.00271-18 30834331 PMC6392095

[51] Tojo R Suárez A Clemente M de los Reyes-Gavilán C Margolles A Gueimonde M Intestinal microbiota in health and disease: role of *Bifidobacteria* in gut homeostasis. *World J Gastroenterol.* (2014) 20:15163. 10.3748/wjg.v20.i41.15163 25386066 PMC4223251

[52] Kearney J. Food consumption trends and drivers. *Philos Trans R Soc B.* (2010) 365:2793–807. 10.1098/rstb.2010.0149 20713385 PMC2935122

[53] Jakicic J Davis K. Obesity and physical activity. *Psychiatr Clin.* (2011) 34:829–40. 10.1016/j.psc.2011.08.009 22098807

[54] Segata N. Gut microbiome: westernization and the disappearance of intestinal diversity. *Curr Biol.* (2015) 25:R611–3. 10.1016/j.cub.2015.05.040 26196489

[55] Rampelli S Schnorr S Consolandi C Turroni S Severgnini M Peano C Metagenome sequencing of the Hadza hunter-gatherer gut microbiota. *Curr Biol.* (2015) 25:1682–93. 10.1016/j.cub.2015.04.055 25981789

[56] Fouesnard M Zoppi J Pétéra M Le Gleau L Migné C Devime F Dietary switch to Western diet induces hypothalamic adaptation associated with gut microbiota dysbiosis in rats. *Int J Obes.* (2021) 45:1271–83. 10.1038/s41366-021-00796-4 33714973

[57] Rios-Covian D González S Nogacka A Arboleya S Salazar N Gueimonde M An overview on fecal branched short-chain fatty acids along human life and as related with body mass index: associated dietary and anthropometric factors. *Front Microbiol.* (2020) 11:973. 10.3389/fmicb.2020.00973 32547507 PMC7271748

[58] Liu F Li P Chen M Luo Y Prabhakar M Zheng H Fructooligosaccharide (FOS) and galactooligosaccharide (GOS) increase *Bifidobacterium* but reduce butyrate producing bacteria with adverse glycemic metabolism in healthy young population. *Sci Rep.* (2017) 7:11789. 10.1038/s41598-017-10722-2 28924143 PMC5603605

[59] Brasili E Hassimotto N Del Chierico F Marini F Quagliariello A Sciubba F Daily consumption of orange juice from citrus *Sinensis* L. Osbeck cv. cara cara and cv. Bahia differently affects gut microbiota profiling as unveiled by an integrated meta-omics approach. *J Agric Food Chem.* (2019) 67:1381–91. 10.1021/acs.jafc.8b05408 30644740

[60] Martin del Campo F Vega Magaña N Salazar-Félix N Peña Rodríguez M Romo-Flores M Cortés Sanabria L P1144 Odoribacter and *Anaerotruncus*: gut microbiome signature might be related to cognitive impairment in patients on peritoneal dialysis. *Nephrol Dial Transplan.* (2020) 35 (Suppl. 3):gfaa142.P1144. 10.1093/ndt/gfaa142.P1144

[61] Zhuang P Zhang Y Shou Q Li H Zhu Y He L Eicosapentaenoic and docosahexaenoic acids differentially alter gut microbiome and reverse high-fat diet–induced insulin resistance. *Mol Nutr Food Res.* (2020) 64:1900946. 10.1002/mnfr.201900946 32298529

[62] Zhao Q Hou D Fu Y Xue Y Guan X Shen Q. Adzuki bean alleviates obesity and insulin resistance induced by a high-fat diet and modulates gut microbiota in mice. *Nutrients.* (2021) 13:3240. 10.3390/nu13093240 34579118 PMC8466346

[63] Vacca M Celano G Calabrese F Portincasa P Gobbetti M De Angelis M. The controversial role of human gut *Lachnospiraceae*. *Microorganisms.* (2020) 8:573. 10.3390/microorganisms8040573 32326636 PMC7232163

[64] Huang F Hong R Yi Y Bai Y Dong L Jia X *In vitro* digestion and human gut microbiota fermentation of *Longan* pulp polysaccharides as affected by *Lactobacillus fermentum* fermentation. *Int J Biol Macromol.* (2020) 147:363–8. 10.1016/j.ijbiomac.2020.01.059 31923510

